# Very Low Birth Weight Infant Necessitating Nissen Fundoplication for Weaning off the Mechanical Ventilator 

**Published:** 2014-05-21

**Authors:** İpek Güney Varal, Nilgün Köksal, Hilal Özkan, Pelin Doğan, Onur Bağcı, Hasan Doğruyol, Arif Gürpınar

**Affiliations:** 1 Uludağ University, Faculty of Medicine, Department of Pediatrics, Division of Neonatology, Turkey;; 2 Uludağ University, Faculty of Medicine, Department of Pediatric Surgery, Turkey;

**Keywords:** Nissen fundoplication, Gastro-esophageal reflux, Premature

## Abstract

Gastro-esophageal reflux (GER) is one of the common problems of neonatal intensive care units. Although this condition does not always need to be treated, it occasionally causes clinically serious consequences. Initial management is medical; however, in some cases surgery might be required. A premature neonate with birth weight of 1370 grams was managed in our ICU. The patient was mechanical ventilator dependent due to GER. The patient needed Nissen fundoplication for successfully weaning off the ventilator.

## INTRODUCTION

GER is a well-recognized condition in newborn infants which resolves with physiological maturation of lower esophageal sphincter and lengthening of intra-abdominal esophagus in the first few months of life. GER represents a common condition in preterm infants. Reflux may manifest as vomiting or be implicated as a silent cause of aspiration pneumonia, apnea, bradycardia, and rarely sudden infant death syndrome. In some infants, the management of GER may be difficult especially who are mechanical ventilator dependent and recurrent aspiration pneumonia preclude weaning off. Medical therapy includes milk/formula thickeners, head-up positioning, prokinetic agents, and H2 receptor antagonists, and is effective in majority of cases.[1] Fundoplication is required in symptomatic infants with GER that does not respond to medical treatment. Few cases are reported of antireflux surgery for the neonatal intensive care-dependent infants.[2,3] We report a case of a very low birth weight (VLBW) premature infant, who had severe respiratory problems that needed mechanical ventilation despite all medical therapy, and demonstrated dramatic clinical and radiological improvement after Nissen fundoplication.

## CASE REPORT

A 1370-gram and 30-week-old infant was delivered with APGAR scores 4-6 via urgent cesarean section due to acute fetal distress. The infant was given surfactant treatment due to respiratory distress syndrome and required mechanical ventilation. Patent ductus arteriosus was found on echocardiography and three day ibuprofen treatment was given for that. Over 2-3 weeks, the patient showed respiratory distress on NG feeding consistent with GER. Feedings were attempted in head-up position with condensed formula but no response found. X-ray chest showed pneumonic infiltration with atelectasis. pH metery was performed which confirmed GER. Sodium alginate and domeperidone treatment was initiated; trans-pyloric tube feeding was also attempted but remained in-vain. The patient was continued on mechanical ventilated due to recurrent aspiration pneumonia. Therefore, surgical treatment was planned and Nissen fundoplication was performed on 60th day of hospitalization. No complication developed following the operation. The patient was extubated and started enteral feeding on the 3rd postoperative day. In a week after surgery full oral feeding was achieved. The patient was discharged in good clinical condition on 10th postoperative day.

## DISCUSSION

GER is a common problem particularly in premature babies. Continuous intraluminal esophageal pH monitoring can be used to quantify the frequency and duration of GER.[4] Intraluminal electrical impedance monitoring and contrast agent radiology can be considered among other methods. Medical treatment for suspected GER is in NICU is common. We initially tried medical treatment in our patient. Furthermore, feeding by trans-pyloric tube (TPT) was also tried. Surgery must be considered if there is insufficient weight gain despite treatment, recurrent aspiration pneumonia, anemia related to bleeding, persistent vomiting, and stricture formation.

In Neonatal ICU patients, especially premature and low birth weight along with other comorbidities, Nissen fundoplication and gastrostomy placement are technically challenging. Nissen fundoplication was performed in our case due to recurrent vomiting, aspiration and failure of weaning off the ventilator. The patient was weaned off the mechanical ventilator on the third postoperative day. Barnes et al [1] reported longer duration to establish oral feeding in their cases. Macharia et al [5] performed fundoplication in a group of patients with the aim of improving respiratory status and weaning off the ventilation. The median age at surgery was 5.8 months and the median weight at surgery was 6.3 kg. In our case, VLBW infant (1370 gr) was operated at the age of 7 weeks and weight of 1900 g.

In conclusion, though surgery is not the first choice in the treatment of GER, in some mechanical ventilator depended cases, it is indispensable.

## Footnotes

**Source of Support:** Nil

**Conflict of Interest:** None declared

